# Enhanced Energy Storage Capacity in NBT Micro-Flake Incorporated PVDF Composites

**DOI:** 10.3390/polym17111486

**Published:** 2025-05-27

**Authors:** Tingwei Mei, Mingtao Zhu, Hongjian Zhang, Yong Zhang

**Affiliations:** 1 State Key Laboratory of Advanced Technology for Materials Synthesis and Processing, School of Materials Science and Engineering, Wuhan University of Technology, Wuhan 430070, China; 331043@whut.edu.cn (T.M.); 331226@whut.edu.cn (M.Z.); zhanghongjian@whut.edu.cn (H.Z.); 2 Center for Smart Materials and Device Integration, Wuhan University of Technology, Wuhan 430070, China

**Keywords:** energy storage, sodium bismuth titanate, charge–discharge, power density

## Abstract

In recent years, dielectric films with a high energy-storage capacity have attracted significant attention due to their wide applications in the fields of renewable energy, electronic devices, and power systems. Their fundamental principle relies on the polarization and depolarization processes of dielectric materials under external electric fields to store and release electrical energy, featuring a high power density and high charge–discharge efficiency. In this study, sodium bismuth titanate (NBT) micro-flakes synthesized via a molten salt method were treated with hydrogen peroxide and subsequently blended with a polyvinylidene fluoride (PVDF) matrix. An oriented tape-casting process was utilized to fabricate a dielectric thin film with enhanced energy storage capacity under a weakened electric field. Experimental results demonstrated that the introduction of modified NBT micro-flakes facilitated the interfacial interactions between the ceramic fillers and polymer matrix. Additionally, chemical interactions between surface hydroxyl groups and fluorine atoms within PVDF promoted the phase transition from the α to the β phase. Consequently, the energy storage density of PVDF-NBT composite increased from 2.8 J cm^−3^ to 6.1 J cm^−3^, representing a 110% enhancement. This design strategy provides novel insights for material innovation and interfacial engineering, showcasing promising potential for next-generation power systems.

## 1. Introduction

Dielectric energy storage represents a critical research frontier in advanced capacitor technologies [1], with primary objectives centered on achieving high energy density [2], charge–discharge efficiency, and long-term reliability through optimization of material and structural designs [3]. The energy storage performance [4,5,6] of dielectric films is predominantly determined by dielectric permittivity, breakdown strength, and polarization characteristics [7]. This has driven extensive exploration of hybrid systems combining high-permittivity materials (e.g., barium titanate-based [8] and zirconia-based [9] compositions, etc.) with high-breakdown configurations [10,11,12,13] such as multilayered heterointerfaces [14] and nanocomposite architectures [15,16,17]. Recent advances have demonstrated that strategic approaches—including interface engineering [18], defect modulation [19], and nanofiller [20] incorporation (e.g., graphene and boron nitride nanosheets)—can substantially enhance both energy storage density [21] and charge–discharge efficiency [22,23,24]. In this work, we incorporated the modified NBT micro-flakes into PVDF. With the aim of developing a flexible energy storage material that performs well under low electric fields, this study provides valuable insights into the development of this field.

Yue [25] et al. reported enhanced energy storage performance up to 6.5 J/cm^3^ by incorporating BaTiO_3_ into PVDF with PMMA as a reinforcement phase. Similarly, Shraddha [26] et al. improved the overall energy storage density by co-doping PZT and BNT into PVDF, which dramatically increased the β phase content. This mechanism of enhancing the β phase through ceramic doping has inspired our work. Tang [27] have revealed that incorporating high-aspect-ratio BaTiO_3_ nanowires (NWs) into a PVDF matrix significantly enhances dielectric permittivity compared to low-aspect-ratio nanoparticles (NPs). Zhu [28] employed finite element simulations to analyze electric field distribution in PVDF-HFP composites containing TiO_2_ nanostructures, of which TiO_2_ nanosheets induce more homogeneous field distribution than nanoparticles or nanorods [29] through planar interface-induced directional charge diffusion. Yao [30] developed PVDF-based composites by introducing polydopamine modified SrTiO_3_ fibers (ST-PDA). With 5 vol% ST-PDA fibers, the breakdown strength reached 3600 kV/cm, corresponding to a discharge energy density of 9.12 J cm^−3^ (190% enhancement versus pristine PVDF). Zhao [31] implemented hot-pressing technology to construct multilayer PVDF/FKM-GNP/PVDF structures. This architecture exhibited a discharge energy density of 6.1 J cm^−3^ at 3200 kV/cm, attributed to enhanced β phase content through thermal-induced α to β phase transition.

In this study, molten-salt-derived Na_0.5_Bi_0.5_TiO_3_ micro-flakes (NBT) were surface-modified with hydrogen peroxide to achieve two objectives: (1) improving dispersion homogeneity in the PVDF matrix; (2) strengthening interfacial bonding through hydroxyl-F interactions. These modifications synergistically elevate breakdown strength while maintaining a high polarization response, establishing a new paradigm for interface-engineered dielectric composites. Despite recent progress, critical challenges persist in simultaneously enhancing breakdown strength, improving charge–discharge efficiency, ensuring thermal stability under elevated temperatures, and developing scalable manufacturing processes for large-scale dielectric films [32,33,34,35]. This study proposes a strategy for developing high-performance dielectric composites through multiscale interface optimization, demonstrating promising potential for emerging applications in energy storage systems.

## 2. Experimental Section

### 2.1. Materials

Titanium(IV) oxide (TiO_2_, ≥99%, Aladdin, Shanghai, China), barium carbonate (BaCO_3_, ≥99%, Aladdin, Shanghai, China), bismuth oxide (Bi_2_O_3_, ≥99%, Aladdin, Shanghai, China), sodium carbonate (NaCO_3_, ≥99%, Aladdin, Shanghai, China), potassium chloride (KCl, ≥99%, Aladdin, Shanghai, China), sodium chloride (NaCl, ≥99%, Aladdin, Shanghai, China), N,N-dimethylformamide (DMF, ≥99.5%, Aladdin, Shanghai, China), poly(vinylidene fluoride) (PVDF, -(CH_2_CF_2_)_n_-, Aladdin, Shanghai, China), and absolute ethanol (CH_3_CH_2_OH, ≥99.8%, Aladdin, Shanghai, China) were used as received. All chemicals were stored in a nitrogen-filled glovebox prior to use.

### 2.2. Ceramic Filler Fabrication

Sodium bismuth titanate micro-platelets were synthesized via molten salt synthesis following three-stages of phase evolution:

(1) $$2{Bi}_{2}O_{3}+3{TiO}_{2}\overset{NaCl\;KCl}{\to }{Bi}_{4}{Ti}_{3}O_{12}$$

(2) $${Bi}_{4}{Ti}_{3}O_{12}+{2Na}_{2}{CO}_{3}+5{TiO}_{2}\overset{NaCl\;KCl}{\to }8{Na}_{0.5}{Bi}_{4.5}{Ti}_{4}O_{15}+2CO_{2}↑$$

(3) $${Na}_{0.5}{Bi}_{4.5}{Ti}_{4}O_{15}+2{Na}_{2}CO_{3}+5TiO_{2}\overset{NaCl\;KCl}{\to }{(Na}_{0.5}{Bi}_{0.5})TiO_{3}+2CO_{2}↑$$

First, the bismuth-layered bismuth titanate micro-platelets were synthesized [36], and then the Bi in the micro-platelets was replaced with Na via a chemical flipping method [37]. In this substitution process, the material undergoes a series of reactions in the molten salt, including the transformation of the crystal from the bismuth-layered structure to the perovskite structure. The thickness of sodium bismuth titanate micro-platelets can be modulated by adjusting the appropriate temperature and holding time.

### 2.3. Film Fabrication

**Figure 1:**
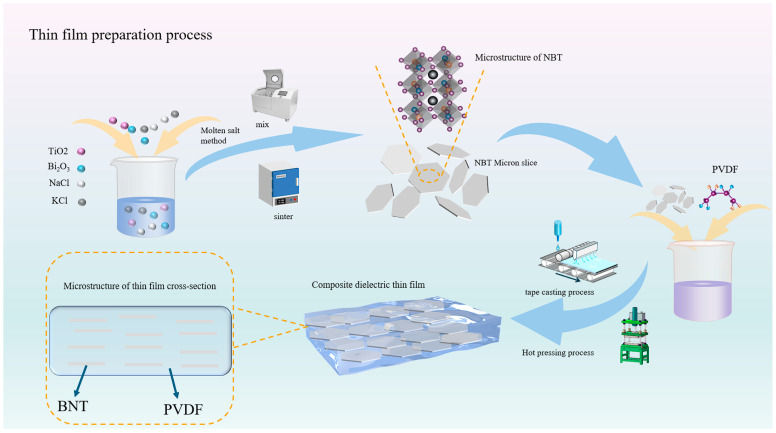
Preparation of NBT micro-flakes and PVDF-based dielectric film.

To prepare PVDF films, 0.2 g of PVDF powder was dissolved in 4 mL of DMF solution. The mixture was magnetically stirred at ambient temperature for 12 h to ensure complete dissolution. Ceramic fillers were then added at concentrations of 1 wt%, 3 wt%, and 5 wt%, with a blank control group established. The solution was stirred and ultrasonicated prior to casting to guarantee uniform dispersion of solutes. Using a doctor-blade process, the PVDF film was cast onto a glass substrate, with the blade gap height set as 20 μm. All films were placed in a drying oven at 60 °C for 24 h to fully evaporate the organic solvent. The dried films were hot-pressed at 110 °C (pressure ~20 MPa) for 30 min, ultimately yielding PVDF-based films with defined dimensions. The preparation process is illustrated in Figure 1.

### 2.4. Material Characterization

Microstructural analysis was conducted using field-emission scanning electron microscopy (FE-SEM, JSM-7610FPlus, JEOL, Tokyo, Japan) operated at 5 kV. Prior to imaging, samples were sputter-coated with a 5 nm Au/Pd layer to enhance conductivity. Elemental mapping was performed on cross-sectional specimens using a Zeiss Ultra Plus SEM equipped with Oxford X-MaxN 80 EDS detector. Crystalline phase identification was carried out via X-ray diffraction (XRD, SmartLab, Rigaku, Tokyo, Japan) with Cu K_α_ radiation (λ = 0.15418 nm) at 2°/min scanning rate. Residual stress analysis was derived from peak shift quantification using the Williamson–Hall method.

### 2.5. Energy Storage Performance

Energy storage properties were evaluated using a precision ferroelectric tester (Radiant Premier II, Albuquerque, NM, USA) with a 10 kV HVI-SC module. Samples immersed in silicone oil were subjected to unipolar electric fields (10 Hz sinusoidal waveform) up to 4000 KV/cm. Polarization-electric field (D-E) hysteresis loops were recorded for energy density calculation via numerical integration [33,38]:

(4) $$U=\int_{D_{r}}^{D_{max}}E\;dD$$

(5) $$U_{0}=\int_{0}^{D_{max}}E\;dD$$

The key parameters are defined as follows: where *E* is the electric field and *D* is the electric displacement [39].

The charge–discharge efficiency can be calculated according to the following formula:

(6) $$\eta =\frac{U}{U_{0}}$$

where *η* represents the charge–discharge efficiency, *U* is the discharge energy density, and *U*_0_ denotes the total charged energy density [40].

### 2.6. Calculation of β Phase

In this study, the conformation and crystal phase of the polymer chain were determined by Nexus 670 Fourier transform infrared spectrometer (FTIR) (Thermo Fisher, Waltham, MA, USA). According to Lambert–Beer’s law, Equation (7) is derived, and the relative fraction of β phase is calculated, where A_α_ and A_β_ are the absolute intensities of the peaks at 763 cm^−1^ and 841 cm^−1^, respectively [41,42,43]:

(7) $$F\left(\beta \right)=\frac{X_{\beta }}{\left(X_{\alpha }+X_{\beta }\right)}=\frac{A_{\beta }K_{\alpha }}{K_{\beta }A_{\alpha }+K_{\alpha }A_{\beta }}\times 100\%$$

*K_α_* and *K_β_* represent the absorption coefficients at each wavenumber, which are 6.1 × 10^4^ cm^2^/mol and 7.7 × 10^4^ cm^2^/mol, respectively [44].

## 3. Results and Discussion

**Figure 2:**
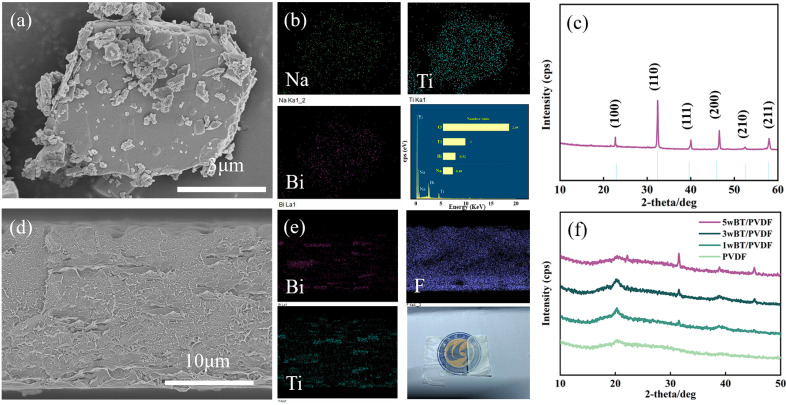
(**a**) SEM image of the NBT micro-flakes; (**b**) EDS spectra of the NBT micro-flakes; (**c**) X-ray diffraction pattern of NBT micro-flakes; (**d**) SEM image of the film cross-section; (**e**) EDS spectra of the film cross-section; (**f**) X-ray diffraction pattern of PVDF-NBT composites with different doping concentrations.

Figure 2a presents the overall flaky morphology of NBT fillers, with a relatively smooth surface. The thickness of NBT is about 0.3 μm, which proves that NBT micro-flakes were successfully synthesized via the molten salt method. The even distribution of Na, Ti, and Bi elements (atomic ratio of 0.5:0.5:1:3) in NBT micro-flakes was confirmed by Figure 2b. As can be seen in Figure 2c, the NBT micro-flakes have a perovskite cubic crystal structure and no impurity could be detected. Figure 2d displays the cross-sectional morphology of PVDF-based composite doped with 3 wt% NBT. A uniform and smooth interface is observed. Distinct arrangements of NBT micro-flakes in PVDF matrix can be seen from the cross-sectional morphology (Figure 2d), and can be further determined through the elemental distribution (Figure 2e). Figure 2f compares the XRD patterns of composite films with different NBT doping concentrations. It can be seen that after adding NBT, two characteristic peaks (32° and 45°) of NBT appeared. As the concentration of NBT micro-flakes increases, the intensity of the characteristic diffraction peaks of NBT gradually increases. This confirms that NBT fillers with different concentrations were indeed incorporated into PVDF.

**Figure 3:**
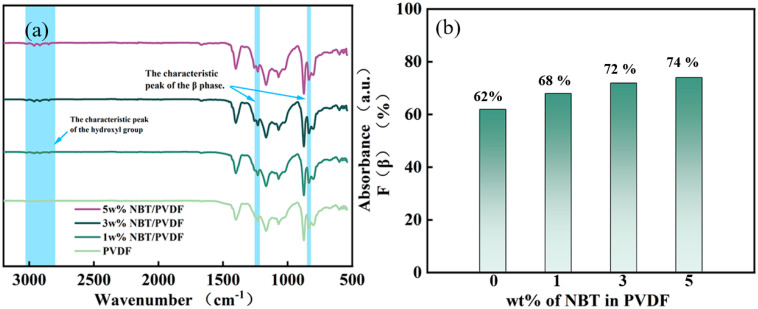
(**a**,**b**) FT−IR spectra and corresponding β phase content with different NBT concentrations.

Figure 3 shows the FT-IR spectra of the PVDF-NBT composites and the corresponding β phase content is given. As calculated by Formula (7), the β phase contents of the PVDF-NBT composites are 62%, 68%, 72%, and 74%, respectively. With 5 wt% NBT doping in PVDF, the β phase content presents an increase of 12% accordingly. This is because NBT can act as a nucleating agent in PVDF, promoting the transformation from the α to the β phase [26]. In addition, the hydroxyl groups attached to the surface of NBT form hydrogen bonds with the fluorine atoms in PVDF, causing the polymer chains to twist, thereby further increasing the β phase content [45]. Therefore, the addition of modified NBT can effectively increase the β phase content of PVDF, and the increase in the β phase helps to increase the energy storage capacity [46].

**Figure 4:**
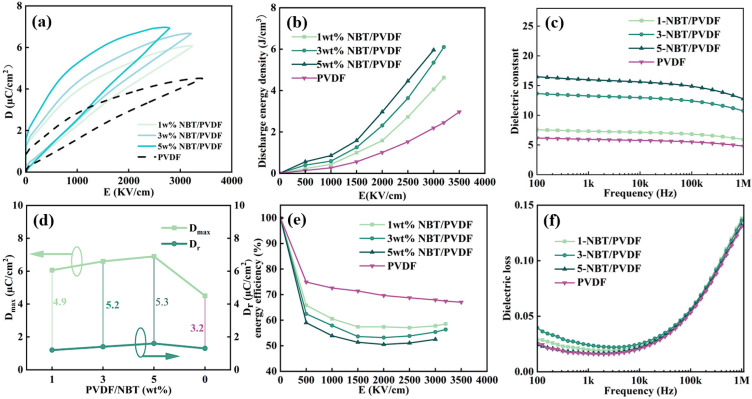
Electrical characteristics of PVDF-NBT composites: (**a**) unipolar D-E hysteresis loops; (**b**) energy storage density; (**c**) dielectric constant; (**d**) D_max_ and D_r_; (**e**) charge–discharge efficiency; (**f**) dielectric Loss.

Figure 4a presents the unipolar D-E hysteresis loops of various PVDF-NBT films. As NBT is added, the electric displacement increases gradually, indicating the optimized ferroelectric properties (Figure 4d). The energy storage performance improves gradually with NBT addition. Figure 4b shows the discharge energy density of various PVDF-NBT films at the highest breakdown voltage. When the mass fraction of NBT is 3 wt%, the discharge energy density reaches up to 6.1 J/cm^3^ at a voltage of 3200 V, which is more than twice that of pure PVDF (2.8 J/cm^3^). The charge–discharge efficiency is above 50%, as can be seen in Figure 4e. When the concentration of NBT is too high, it will lead to the mutual overlap of micro-flakes or their non-parallel arrangement. This causes more defects at the contact areas between NBT and PVDF, resulting in an increase in the dielectric loss [47,48]. Moreover, it becomes more difficult for the domains to reverse and return to their original states, which further affects the charge–discharge efficiency. Figure 4c,f show the dielectric constant and dielectric loss of various composite films. With the increase in NBT concentration, the dielectric properties of the films are greatly improved. Compared with the pure PVDF, the dielectric constant can reach the highest value of 20 after proper NBT incorporation, which is nearly 2.5 times that of pure PVDF.

**Figure 5:**
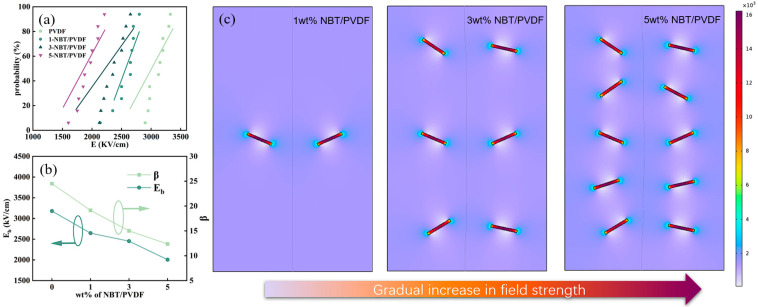
(**a**) Weibull distribution of various PVDF-NBT composites; (**b**) corresponding shape factor and breakdown voltage; (**c**) simulation result.

Figure 5a shows the Weibull distribution of various PVDF-NBT composites; the breakdown electric field decreases with the increased NBT concentration. Considering the relatively low breakdown field strength of NBT ceramics, multiple NBT stacks inside PVDF can easily form a through-thickness breakdown channel from top to bottom [49]. Therefore, the breakdown strength is lower at high NBT concentrations. Combined with the shape factor in Figure 5b, it can be found that as the NBT concentration increases, the breakdown electric field decreases from 3500 kV/cm to 2500 kV/cm, and the shape factor decreases from 27 to 15. This indicates that with the increase in NBT, the stability of the film decreases, and this phenomenon mainly originates from two mechanisms. Firstly, the breakdown strength of NBT ceramic itself is significantly lower than that of PVDF matrix. When the concentration of NBT increases in the composite, it will cause a change in the internal electric field, and the electric field in the middle part will become lower. This structure will make the upper and lower interfaces easier to penetrate. Secondly, high concentrations of NBT can lead to an increased contact area between the two phases, resulting in the formation of more interfaces. Defects and the local concentrated electric field that exist at these interfaces can further reduce the breakdown strength. It is worth noting that although the introduction of NBT can reduce E_b_, the contribution of NBT to polarization improves the energy storage performance. Therefore, the use of an appropriate concentration of NBT doping can enhance the energy storage performance of the composite. Thus, the doping of NBT forms distorted electric field regions, thereby reducing the breakdown electric field. But at the same time, NBT doping can also promote the flipping of domains, thereby improving the energy storage performance. As shown in Figure 5c, COMSOL Multiphysics 5.6 software was used to simulate the internal electric field distribution in NBT/PVDF composite films. The dielectric constant of pure PVDF and NBT was set as 8 and 2000, and the electric field was set as 3000 V/cm [50,51]. It can be observed that at a concentration of 5 wt%, the film will form a large range of electric field distortion areas (blue area) in the film cross-section. In this distorted electric field area, the film is easily broken down. Through the distorted electric field, a vertically connected conductive channel is formed, causing the film to be broken down. The electric field distribution at low concentrations is uniform, and the electric field at most positions is in a stable state. In this work, the PVDF-NBT with 3 wt% NBT doping obtained the best energy storage density, up to 6.1 J cm^−3^.

## 4. Conclusions

In this study, the regulation mechanism of NBT micro-flakes on the structure and properties of PVDF-based dielectric films was systematically discussed. The correlation between NBT concentration and energy storage properties was revealed by multi-scale characterization, and the optimal preparation process was determined. By incorporating the modified NBT into PVDF, the hydroxyl group attached to the surface of NBT interacts with the fluorine atom group inside PVDF and thus increases the β phase content. This method enhances the energy storage capacity from 2.8 J cm^−3^ to 6.1 J cm^−3^, which shapes a new design strategy for performance optimization.

## Data Availability

All data generated or analyzed during this study are included in this published article.
